# Detection of Acute and Early HIV-1 Infections in an HIV Hyper-Endemic Area with Limited Resources

**DOI:** 10.1371/journal.pone.0164943

**Published:** 2016-10-20

**Authors:** Simnikiwe H. Mayaphi, Desmond J. Martin, Thomas C. Quinn, Oliver Laeyendecker, Steve A. S. Olorunju, Gregory R. Tintinger, Anton C. Stoltz

**Affiliations:** 1 Department of Medical Virology, University of Pretoria, City of Tshwane, South Africa; 2 National Health Laboratory Service-Tshwane Academic Division (NHLS-TAD), City of Tshwane, South Africa; 3 Toga Laboratories, Johannesburg, South Africa; 4 Division of Intramural Research, National Institute of Allergy and Infectious Diseases, National Institutes of Health, Bethesda, Maryland, United States of America; 5 Johns Hopkins University School of Medicine, Baltimore, Maryland, United States of America; 6 Biostatistics unit, Medical Research Council, City of Tshwane, South Africa; 7 Department of Internal Medicine, University of Pretoria, City of Tshwane, South Africa; Waseda University, JAPAN

## Abstract

**Background:**

Two thirds of the world’s new HIV infections are in sub-Saharan Africa. Acute HIV infection (AHI) is the time of virus acquisition until the appearance of HIV antibodies. Early HIV infection, which includes AHI, is the interval between virus acquisition and establishment of viral load set-point. This study aimed to detect acute and early HIV infections in a hyper-endemic setting.

**Methods:**

This was a cross-sectional diagnostic study that enrolled individuals who had negative rapid HIV results in five clinics in South Africa. Pooled nucleic acid amplification testing (NAAT) was performed, followed by individual sample testing in positive pools. NAAT-positive participants were recalled to the clinics for confirmatory testing and appropriate management. HIV antibody, p24 antigen, Western Blot and avidity tests were performed for characterization of NAAT-positive samples.

**Results:**

The study enrolled 6910 individuals with negative rapid HIV results. Median age was 27 years (interquartile range {IQR}: 23–31). NAAT was positive in 55 samples, resulting in 0.8% newly diagnosed HIV-infected individuals (95% confidence interval {CI}: 0.6–1.0). The negative predictive value for rapid HIV testing was 99.2% (95% CI: 99.0–99.4). Characterization of NAAT-positive samples revealed that 0.04% (95% CI: 0.000–0.001) had AHI, 0.3% (95% CI: 0.1–0.4) had early HIV infection, and 0.5% (95% CI: 0.5–0.7) had chronic HIV infection. Forty-seven (86%) of NAAT-positive participants returned for follow-up at a median of 4 weeks (IQR: 2–8). Follow-up rapid tests were positive in 96% of these participants.

**Conclusions:**

NAAT demonstrated that a substantial number of HIV-infected individuals are misdiagnosed at South African points-of-care. Follow-up rapid tests done within a 4 week interval detected early and chronic HIV infections initially missed by rapid HIV testing. This may be a practical and affordable strategy for earlier detection of these infections in resource-constrained settings. Newer molecular tests that can be used at the points-of-care should be evaluated for routine diagnosis of HIV in hyper-endemic settings.

## Introduction

Halting and reversing the spread of HIV was part of Millennium Development Goal (MDG) 6A [1]. Although good progress has been made in achieving MDG 6A, there were still too many new HIV infections by the end of 2014 and 2015, and two thirds of these infections were found in sub-Saharan Africa [2,3]

Acute HIV infection (AHI) refers to the time of virus acquisition until the appearance of HIV antibodies. Early or primary HIV infection, which includes AHI, is regarded as the interval between virus acquisition and the establishment of HIV viral load (VL) set-point [4]. Chronic HIV stage follows after the set-point is established [5]. People with early HIV infection contribute significantly to the transmission of HIV, as they have very high VLs in blood and genital secretions. It is estimated that early HIV infection stage is 26 times more infectious compared to the chronic stage [6]. This early stage of HIV infection is also known to predominantly produce C-C chemokine receptor type 5 (CCR-5) HIV strains, which are efficiently transmitted across the genital mucosa [7].

Rapid HIV tests play a crucial role in detecting HIV infections, and thereby initiating a cascade of linking infected patients to care. These rapid tests are commonly used for diagnosis of HIV infection in low resource settings such as in sub-Saharan Africa, but have poor sensitivity for detection of early HIV infection [8,9], which results in giving false negative results to highly infectious individuals. The addition of p24 antigen to some rapid HIV tests has led to a slight improvement in sensitivity for detection of early HIV infections, as the p24 antigen component on these tests performs poorly [8,9]. Tests that have shortened the HIV window period such as enzyme-linked immunosorbent assays (ELISAs) and NAATs are costly and not readily available for point-of-care testing [10].

Management of early HIV infection has benefits for the infected individual, and prevents secondary spread of HIV in the population [4,5]. This study aimed to detect acute and early HIV infections in an HIV hyper-endemic setting with limited resources.

## Materials and Methods

### Recruitment and enrollment

This was a cross-sectional diagnostic study, conducted between March 2012 to June 2015, which enrolled individuals who had negative rapid HIV results and were 14 years or older. Participants were recruited and enrolled from 5 HIV counseling and testing (HCT) clinics in the Tshwane district of South Africa (SA). Four of these HCT clinics were antenatal clinics and one was a general HCT clinic. Rapid HIV testing was done according to the SA HIV testing guidelines, which recommend a serial HIV testing strategy at the points-of-care [11]. Testing at the HCT clinics was done by HIV counselors, who had received training in HIV testing and counseling. The rapid HIV test that was commonly used for screening during the course of this study was Advanced Quality (Intec Products Inc). Abon (Abon Biopharm) was used for screening in 2014; however, this was replaced with Advanced Quality at the end of 2014. At enrolment, study samples and participants’ cell phone numbers were collected. Plasma was separated from whole blood through centrifugation at 1700 relative centrifugal force (RCF) for 20 minutes, and stored at -70°C within 24 hours after collection until the time of testing.

### Ethics statement

Written consent was obtained from all participants before enrollment. The study was approved by the University of Pretoria’s Faculty of Health Sciences Ethics Committee (Protocol number– 295/2015) and by Tshwane Research Ethics Committee (TMREC 2010/26). The legal ages for consenting to HIV testing and medical treatment in South Africa are 12 and 14 years, respectively [12]. During the course of the study we noticed that some people who came for HIV testing were younger than 18 years of age, and came alone without parents or guardians. Hence, we amended our study protocol to include this group, in order to extend the benefit of earlier diagnosis of HIV to them. We applied for inclusion of this group with our Ethics Committee, and were granted approval. So all study participants, including 14–17 years, signed the same written consent forms that were used for participants older than 18 years.

### Sample testing

Roche CAP/CTM HIV VL version 2 assay (Roche Diagnostics, Mannheim, Germany) with a lower detection limit of 20 copies/ml was used for NAAT, in a mini-pool of 5 samples, using 200 μl from each sample to constitute a 1 ml sample volume required for testing. An additional volume of about 20 μl from one of the pool samples was used for top-up in order to avoid sample rejection due to insufficient volume. Pools that had undetectable VL were considered negative for HIV. Individual sample testing was done in pools that had detectable VL. A VL threshold of ≥5000 copies/ml in an individual sample was considered as diagnostic for HIV infection. If HIV VL was <5000 copies/ml, a repeat test was done in a follow-up sample in order to exclude a possible contamination in the initial test [5]. All participants were encouraged to voluntarily collect NAAT results; however, those who tested positive on NAAT were contacted on their cell phones to come back to the clinic for further management. During this follow-up visit, HIV counseling and repeat rapid testing were done, follow-up samples collected, and participants referred for appropriate management.

The following serology tests were done in NAAT-positive samples: 3^rd^ generation Genscreen HIV-1/2 version 2 ELISA (BioRad, Marnes-la-Coquette, France) and HIV Western Blot (Bio-Rad Laboratories, Redmond WA, USA) for antibody detection; p24 antigen (Roche Diagnostics, Mannheim, Germany); and limiting antigen (LAg) HIV avidity assay {Maxim Biomedical Inc., Rockville, USA} for confirmation of early HIV infection in samples with detectable antibodies. LAg avidity assay was repeated on follow-up samples. Acute HIV infection was defined as the presence of HIV RNA with or without p24 antigen in the absence of HIV antibodies. Early HIV infection was defined as the presence of HIV RNA with or without p24 antigen, and presence of HIV antibodies with low avidity as reflected by values <1.5 normalised optical density (OD-n) on LAg avidity assay. Samples found to have HIV RNA with or without p24 antigen, and HIV antibodies with high avidity of >1.5 OD-n were classified as having chronic infection.

Rapid HIV testing was later repeated from stored plasma samples in the laboratory using the same tests that were used at the points-of-care. All the tests were performed and analyzed according to manufacturer’s instructions. CD4 count results were later enumerated from the laboratory information system and patient records for NAAT-positive participants.

### Statistical analysis

A descriptive analysis was used to present summary statistics (median, proportions and 95% confidence intervals) for the parameters. This was followed by a comparison between the groups using two sample independent t-tests for proportions. Proportions of acute, early and chronic HIV infections that were missed at the points-of-care were computed. Comparison of the proportions of newly diagnosed HIV infections between males and females was done. In addition, median values of HIV VL and CD4 count were compared between the groups of participants with early and chronic HIV infections. All the statistics were performed on the STATA version 14.1 software (StataCorp LP, College Station, TX, USA). A p-value of ≤0.05 was considered statistically significant.

## Results

### Demographics and newly diagnosed HIV infections

**Fig 1:**
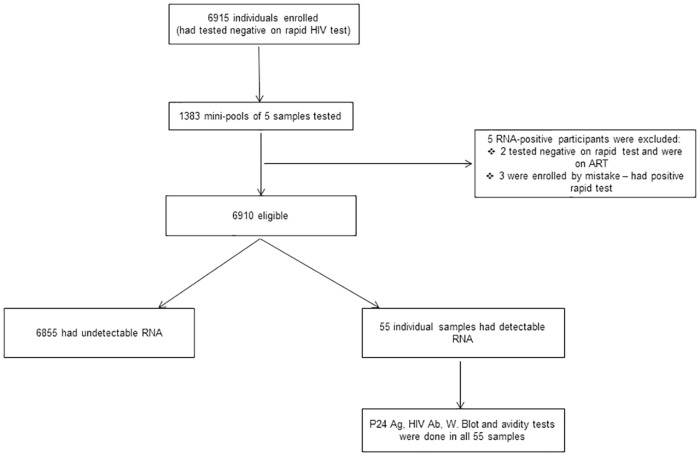
Algorithm showing enrolment and testing of participants. Participants’ baseline rapid results were reviewed if they had positive HIV RNA results. Those who had negative rapid test and receiving antiretroviral therapy, and those enrolled by mistake (positive baseline rapid test) were excluded. Ag = antigen, Ab = antibody, W. Blot = Western Blot.

From March 2012 to June 2015, the study enrolled and tested 6910 participants who had negative rapid HIV test results (Fig 1). Their median age was 27 years (IQR: 23–31). Females formed a large proportion (87%, n = 6011) of the study group, and 88% (n = 5271) of the female participants were pregnant. NAAT detected HIV RNA in 55 samples, resulting in 0.8% of newly diagnosed HIV-infected individuals (95% CI: 0.6–1.0). This showed a negative predictive value (NPV) of 99.2% (95% CI: 99.0–99.4) for rapid HIV testing at the points-of-care. The newly diagnosed HIV infections were detected in all five study clinics, and were slightly higher in pregnant females (0.9%) compared to non-pregnant females and males, 0.7% (p = 0.62) and 0.6% (p = 0.36), respectively.

### Serological characterization of NAAT-positive samples

**Fig 2:**
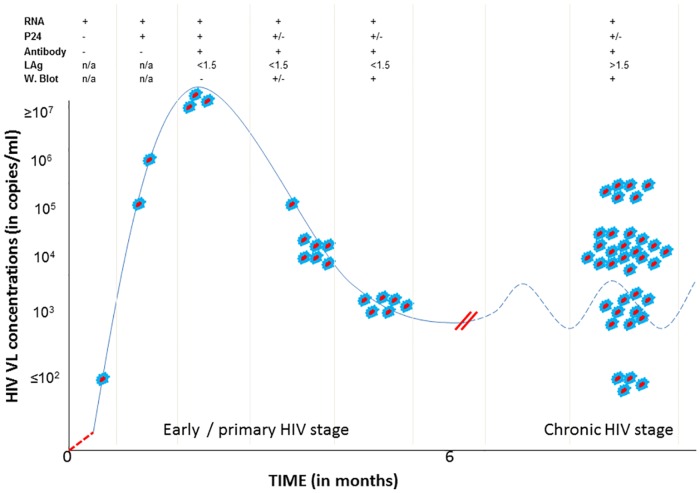
Staging of identified HIV infections based on HIV RNA (viral load), p24 antigen, antibodies, limiting antigen (LAg) avidity results and Western Blot (W. Blot). All the red-blue dots represent participants with HIV infections that were misdiagnosed by rapid HIV tests at the points-of-care. Solid blue line represents HIV viral load during early HIV stage, and dotted blue line represents HIV viral load fluctuation during the chronic stage. Red dotted line represents eclipse stage where all HIV tests are negative. Double red lines represent HIV viral load set-point. Participants with LAg avidity results <1.5 OD-n values had early HIV infections and those with LAg results >1.5 OD-n values had chronic HIV infections. N/A = not applicable, + = positive, - = negative, < = less than, > = greater than, +/- = positive or negative results.

Of the 55 NAAT-positive participants, 52 (95%) tested positive for HIV antibodies on Genscreen HIV ELISA. Western Blot (W. Blot) was positive in 48 (92%) of these antibody-positive participants. P24 antigen testing was performed on 53 participants, and was positive in 12 (23%) participants. The two samples that were insufficient for p24 antigen test were positive on HIV ELISA. Limiting antigen avidity assay identified 16 antibody-positive participants as having early HIV infection, and classified the remainder (n = 36) as having chronic infection. Limiting antigen avidity was repeated on follow-up samples of participants who came for a follow-up visit, and the results remained the same as in initial testing. The combination of HIV RNA, p24 antigen, HIV antibody, LAg avidity and W. Blot test results enabled staging of HIV infections. Amongst those with early HIV infection, few participants were detected very early before the appearance of HIV antibodies, some were detected at the peak of HIV vireamia, and many others were near the time of HIV VL set-point (Fig 2). A significant proportion of HIV-infected individuals were classified as having chronic HIV infection (Fig 2).

Serological characterization of NAAT-positive samples showed that 0.04% (95% CI: 0.000–0.001) had acute HIV infection (n = 3); 0.3% (95% CI: 0.1–0.4) had early HIV infection (n = 19, including those with acute infection) (Table 1); and 0.5% (95% CI: 0.5–0.7) had chronic HIV infection (n = 36) (Table 2).

**Table 1:** Characteristics of participants diagnosed with acute or early HIV infection.

			INITIAL TESTS*						FOLLOW-UP TESTS						
Pt ID	Sex	Marital status	Rapid HIV test	HIV VL	p24 antigen	Genscreen 3^rd^ gen ELISA	W. Blot	LAg avidity	F/U interval (weeks)	F/U Rapid HIV test	F/U HIV VL	F/U LAg avidity	HIV Staging	CD4 count	Pregnancy
9498	F	S	-	509793	+ (10.9)	- (0.05)	n/a	n/a	10	+		n/a	Acute	964	No
9228	M	S	-	94	- (0.3)	- (0.06)	n/a	n/a	10	-	71674	n/a	Acute	384	n/a
8047	M	M	-	1245238	+ (33.8)	- (0.03)	n/a	n/a	2	+		n/a	Acute	---	n/a
9218	F	D	-	55099	- (0.4)	+ (5.23)	+	0.143	10	-		0.103	Early	58	No
2066	F	S	-	1010	- (0.5)	+ (4.28)	+	0.058	7	+	38900	0.338	Early	752	No
8575	F	S	-	93079	+ (1.3)	+ (5.06)	+	0.063	8	+		0.171	Early	668	No
7293	F	S	-	5078	- (0.3)	+ (4.76)	+	0.487	ND	ND		ND	Early	---	Yes
5041	M	S	-	22921300	+ (434.1)	+ (2.92)	-	0.073	ND	ND		ND	Early	---	n/a
9049	F	S	-	16848	- (0.3)	+ (5.06)	+	0.201	3	+		0.397	Early	---	Yes
6638	F	S	-	195105	- (0.3)	+ (4.45)	+	0.256	6	+		0.437	Early	457	Yes
261	M	S	-	84501600	+ (1383.0)	+ (4.06)	-	0.073	9	+		1.094	Early	386	n/a
6512	F	S	-	1763	- (0.3)	+ (4.93)	+	1.312	2	+	1938	1.111	Early	215	Yes
6743	F	S	-	27364	- (0.5)	+ (4.48)	-	0.108	7	+		0.418	Early	638	Yes
6582	F	S	-	6216	INSUF	+ (4.81)	+	0.458	6	+		1.060	Early	818	Yes
6727	F	S	-	4874	+ (2.6)	+ (4.48)	+	0.234	2	+	1970	0.258	Early	706	Yes
6737	F	S	-	2227	- (0.3)	+ (5.95)	+	0.746	ND	ND	ND	ND	Early	---	Yes
7084	F	S	-	337987200	+ (2884.0)	+ (0.17)	-	0.065	4	+		0.153	Early	411	No
2504	F	S	-	37243	- (0.4)	+ (5.95)	+	1.211	2	+		1.391	Early	287	Yes
3469	F	S	-	33274	+ (5.6)	+ (5.52)	+	0.832	9	+		0.763	Early	123	Yes

*Initial tests were done from samples obtained at enrolment (i.e after a negative rapid HIV test result). HIV VL tests (including follow-up) were done first to confirm infection, and all the serology tests were done later. Pt ID = participant identity, F = female, M = male or married, S = Single, D = divorced, gen = generation, ELISA = enzyme-linked immunosorbent assay, W. Blot = Western Blot, LAg = limiting antigen, F/U = follow-up, Insuf = insufficient, ND = not done (participant did not come for follow-up), n/a = not applicable, + = positive, - = negative, --- = not available. Units: HIV VL = copies/ml; p24 antigen = cut-off index (COI); Genscreen ELISA = sample cut-off (S/CO); LAg avidity = normalized optical density (OD-n); CD4 count = cells/μl.

**Table 2:** Characteristics of participants diagnosed with chronic HIV infection.

			INITIAL TESTS*						FOLLOW UP TESTS						
Pt ID	Sex	Marital Status	Rapid HIV test	HIV VL	p24 antigen	Genscreen 3^rd^ gen ELISA	W. Blot	LAg avidity	F/U Interval (weeks)	F/U Rapid HIV test	F/U HIV VL	F/U LAg avidity	HIV Staging	CD4 count	Pregnancy
5054	F	S	-	27820	- (0.29)	+ (4.54)	+	4.246	2	+		3.577	LT	72	Yes
5067	F	S	-	12675	INSUF	+ (4.22)	+	3.317	8	+		2.822	LT	147	Yes
9915	F	M	-	14100	+ (1.36)	+ (5.95)	+	4.175	2	+		3.341	LT	382	Yes
4351	F	S	-	2685	- (0.24)	+ (5.96)	+	3.931	3	+	1248	3.267	LT	---	Yes
639	F	S	-	6579	- (0.25)	+ (4.63)	+	3.853	4	+		3.099	LT	392	Yes
641	F	S	-	70569	- (0.32)	+ (5.23)	+	4.116	4	+		3.366	LT	228	Yes
7959	F	S	-	148776	- (0.16)	+ (4.58)	+	3.835	ND	ND		ND	LT	343	No
8828	F	M	-	41500	- (0.42)	+ (5.95)	+	2.382	14	+		2.849	LT	230	Yes
2678	F	M	-	222853	+ (11.13)	+ (4.69)	+	3.639	4	+		3.135	LT	199	Yes
9895	F	M	-	4880	- (0.29)	+ (4.21)	+	4.305	6	+	7873	3.480	LT	607	Yes
9986	F	S	-	97600	- (0.92)	+ (4.75)	+	3.700	2	+		3.093	LT	160	Yes
843	F	S	-	29712	- (0.29)	+ (4.63)	+	2.252	5	+		2.463	LT	348	Yes
6990	F	S	-	17536	- (0.33)	+ (5.96)	+	3.945	6	+		3.233	LT	269	Yes
2340	F	S	-	14490	- (0.36)	+ (4.63)	+	2.850	ND	ND		ND	LT	---	Yes
6709	F	S	-	53	- (0.34)	+ (4.48)	+	3.162	ND	ND	ND	ND	LT	683	Yes
6748	F	S	-	932	- (0.29)	+ (4.68)	+	4.085	2	+	1707	3.324	LT	---	Yes
6671	F	M	-	14072	- (0.23)	+ (4.48)	+	4.083	3	+			LT	---	Yes
6380	F	S	-	11073	- (0.23)	+ (4.62)	+	4.763	4	+		3.509	LT	385	Yes
6557	F	S	-	614	- (0.33)	+ (4.41)	+	4.049	4	+	265	3.593	LT	353	Yes
6565	F	S	-	5670	- (0.37)	+ (4.32)	+	3.091	3	+		2.969	LT	371	Yes
6509	M	M	-	106713	- (0.34)	+ (4.48)	+	3.640	ND	ND		ND	LT	---	n/a
6596	F	S	-	3873	- (0.36)	+ (5.95)	+	4.385	3	+	1087	3.519	LT	576	Yes
6640	F	M	-	3074	- (0.34)	+ (4.48)	+	2.824	5	+	9887	2.864	LT	407	Yes
6649	F	M	-	21051	- (0.28)	+ (5.95)	+	3.804	2	+		2.867	LT	164	Yes
6738	F	S	-	159539	- (0.37)	+ (5.96)	+	4.384	2	+		ND	LT	682	Yes
1067	F	S	-	1779	- (0.37)	+ (4.75)	+	4.088	2	+	2574	3.470	LT	394	Yes
921	F	M	-	9781	- (0.28)	+ (5.04)	+	4.553	7	+		3.607	LT	469	Yes
3869	F	S	-	217372	- (0.31)	+ (5.52)	+	3.716	4	+		3.264	LT	---	Yes
3912	F	S	-	32008	- (0.29)	+ (5.52)	+	2.314	8	+		2.079	LT	287	Yes
3920	F	S	-	66694	- (0.44)	+ (4.68)	+	4.375	8	+		3.410	LT	306	Yes
3880	F	S	-	7505	- (0.30)	+ (4.62)	+	3.840	3	+		3.206	LT	575	Yes
3935	F	S	-	242663	+ (21.77)	+ (5.95)	+	1.833	8	+		2.157	LT	61	Yes
1117	F	M	-	153	- (0.31)	+ (5.95)	+	3.315	2	+	629	3.249	LT	536	Yes
1121	F	S	-	80287	- (0.34)	+ (5.95)	+	3.634	2	+		3.483	LT	127	Yes
3474	F	S	-	16510	- (0.32)	+ (4.91)	+	2.078	12	+		2.828	LT	675	Yes
1475	F	S	-	44450	+ (1.17)	+ (4.92)	+	4.241	9	+		3.628	LT	255	Yes

*Initial tests were done from samples obtained at enrolment (i.e after a negative rapid HIV test result). HIV VL tests (including follow-up) were done first to confirm infection, and all the serology tests were done later. Pt ID = participant identity, F = female, M = male or married, S = Single, D = divorced, gen = generation, ELISA = enzyme-linked immunosorbent assay, W. Blot = Western Blot, LAg = limiting antigen, F/U = follow-up, Insuf = insufficient, LT = long term infection, ND = not done (participant did not come for follow-up), n/a = not applicable, + = positive, - = negative, --- = not available. Units: HIV VL = copies/ml; p24 antigen = cut-off index (COI); Genscreen ELISA = sample cut-off (S/CO); LAg avidity = normalized optical density (OD-n); CD4 count = cells/μl.

### HIV viral loads and CD4 counts of NAAT-positive participants

Median HIV VL was slightly higher in participants with early HIV infection (4.5 log, IQR: 3.7–5.7) compared to those with chronic infection (4.2 log, IQR: 3.8–4.9) (p = 0.33). Most participants (89%) had HIV VLs >1500 copies/ml. CD4 count results were available for 44 participants. Median CD4 count was significantly higher in participants with early HIV infection (434 cells/μl, IQR: 287–706) compared to those with chronic infection (351 cells/μl, IQR: 228–469) (p = 0.03) (Tables 1 and 2).

### Repeat rapid testing from stored plasma samples

Rapid tests were repeated in 50 frozen antibody-positive plasma samples with sufficient volumes in the laboratory. Advanced Quality rapid tests were clearly positive in 45 samples, low positive in 2 (faint band noticed), and negative in 3 samples. Abon rapid tests were clearly positive in 48 samples, low positive in 1 sample and negative in 1 sample.

### Follow-up of NAAT-positive participants

NAAT-positive participants were recalled to the clinics for follow-up, and they came at different intervals based on their availability. Only 47 (86%) participants came back for follow-up at a median of 4 weeks (IQR: 2–8), and 45 (96%) of them were positive on follow-up rapid tests. Follow-up rapid testing done at the points-of-care improved NPV from 99.2% to 99.8%. Participants were managed according to the SA HIV management guidelines, which recommended immediate initiation of antiretroviral therapy (ART) in pregnant females, and ART eligibility when a CD4 count was <350 cells/μl from 2012–2014 and <500 cells/μl from 2015 [13–16]. Two non-pregnant participants, with confirmed early HIV infection, were still negative on rapid test at a 10 week follow-up interval (Table 1). One of them tested positive on the 4^th^ generation ELISA, had a low CD4 count of 58 cells/μl, and was initiated on ART. The other participant tested positive on a second follow-up rapid test done 5 months later, and was still not eligible for treatment as his CD4 count was 384 cells/μl (in 2012). All participants with chronic HIV infection had positive HIV ELISA, positive W. Blot and LAg avidity results of >1.5 OD-n values, and all were positive on follow-up rapid HIV testing done at the points-of-care (Table 2). NAAT-positive participants were asymptomatic or had no severe illnesses as they came back to collect results, with the exception of one who was admitted with acute retroviral syndrome.

## Discussion

### Frequency of new HIV infections

This study evaluated the detection of early HIV infections in a low resource setting through the use of different HIV assays. The finding of 55 (0.8%) newly diagnosed HIV infections missed by rapid tests shows that a substantial number of HIV-infected individuals are misdiagnosed at the points-of-care in SA. A slightly higher prevalence of these infections in females compared to males was not surprising as HIV prevalence in SA is generally higher in females [17]. The newly diagnosed HIV-infected participants were identified in all the study clinics, highlighting that this could be a bigger problem in SA. Other studies in SA and sub-Saharan African countries have identified individuals with early or chronic HIV infection that were misdiagnosed at the points-of-care [18–23]. This calls for improvement of HIV testing guidelines in HIV hyper-endemic setting in order to facilitate earlier detection of HIV-infected individuals who are misdiagnosed by rapid HIV tests at the points-of-care.

From 2002–2012, SA HIV incidence in people aged 15–49 years ranged between 1.9–2.2% [17]. This maintenance of HIV incidence at around 2% for a 10 year period highlights ongoing transmission of HIV, which is probably driven by highly infectious individuals, particularly those with early HIV infection. Most participants (89%) in this study had HIV viral loads above 1500 copies/ml, which is a known risk factor for sexual transmission of HIV [24]. The 0.3% prevalence of early HIV infections noted in this study is lower compared to a recently published study from SA that showed a prevalence of 1.1% in individuals who were HIV antibody-negative at the point-of-care [21]. This lower prevalence of early HIV infection could be explained by the fact that this study was conducted in an area with a moderate HIV prevalence [17], and at a time where there was higher ART coverage in SA [25].

### Earlier detection of HIV-infected individuals misdiagnosed at the points-of-care

The majority of NAAT-positive participants (86%) were successfully recalled to the clinics for further management, showing that it is feasible to detect and manage participants misdiagnosed by rapid HIV testing at the points-of-care. Participants with early HIV infection were detected in all the Fiebig stages of early HIV infection (Fig 2) [26]. The finding that most participants with early HIV infection were near the time of HIV VL set-point could mean that most do not present for HIV testing close to the time of exposure to HIV (Fig 2). Pooled NAAT has been incorporated into the routine screening for HIV in some parts of developed world in order to identify HIV-infected individuals that are misdiagnosed by the rapid tests, particularly those with early HIV infection [27,28]. The availability of resources in the developed world enables detection of these infections, followed by appropriate HIV management [28]. The pooling strategy reduces the costs of NAAT [28]; however, this strategy is expensive to implement for routine diagnosis in low resource settings.

This study showed that follow-up rapid tests done within a 4 week interval are able to detect individuals with early or chronic HIV infection previously misdiagnosed by rapid HIV tests. It is likely that other participants who came for follow-up later than the 4 week interval would have tested positive on rapid HIV tests if they had presented within this interval (Tables 1 and 2). The exception to this was the 2 participants with early HIV infection who were still negative at 10 weeks follow-up (Table 1). Most HIV testing guidelines used in developing countries recommend a follow-up rapid HIV test to be done 12 weeks later after a negative rapid HIV test [16,29,30]. Implementing a 4 week follow-up interval instead of a 12 week follow-up interval would reduce both HIV diagnostic and transmission window periods. Where possible, the 4 week follow-up interval could be incorporated into the existing 12 week follow-up interval to have a 0–4–12 week rapid testing strategy, which could easily be implemented in a setting of frequent clinic visits such as in antenatal clinics. The current WHO HIV testing guidelines recommend follow-up rapid testing at 4–6 weeks only for a minority of individuals who identify a specific recent suspected exposure to HIV [31]. However, it is very difficult to predict or clinically diagnose early HIV infections [5,10]. Hence, a routine follow-up rapid testing at 4 weeks would be a good surveillance tool for early detection of HIV-infected individuals previously misdiagnosed by rapid HIV tests at points-of-care.

### Alternative strategies for detecting HIV-infected individuals misdiagnosed at the points-of-care

Parallel testing algorithm, where HIV testing at the points-of-care is done on two different rapid strips concurrently, could be considered for improving the sensitivity of rapid HIV testing at points-of-care. This strategy has been reported to have a higher sensitivity [32,33]. However, some data show that serial testing is equivalent to the parallel testing algorithm with an added cost saving benefit [34,35]. Black et al. have identified HIV-infected individuals who were misdiagnosed at the points-of-care despite the use of the parallel testing algorithm [36]. This highlights that parallel testing may not be the best solution for improving HIV testing at the points-of-care. WHO recommends either serial or parallel testing algorithm for HIV testing at points-of-care [37].

The other alternative strategy for detection of HIV-infected individuals misdiagnosed by rapid tests in resource limited countries would be to screen individuals testing negative on rapid HIV test with the 4^th^ generation HIV ELISA. However, HIV ELISA is costly and laboratory-based, and thus may also lead to a high rate of loss to follow-up. Molecular tests are now available for use at the points-of-care. These are not readily available in the developing world owing to high costs [38,39]. If NAAT was done at the points-of-care in this study, those misdiagnosed by the rapid HIV test would have been detected and immediately linked to HIV care, without any issues of loss to follow-up.

### Implications of misdiagnosing HIV infections at the points-of-care

Advantages of making an accurate diagnosis of HIV infection include opportunities to preserve HIV-specific immune responses if treatment is made available early, and prevent secondary transmission in the population [5]. There is now convincing evidence showing the benefits of early treatment of HIV-infected patients regardless of CD4 counts [40]. Misdiagnosing HIV-infected individuals at the points-of-care means that some people do not benefit from early treatment of HIV infection. This could lead to complications that are associated with delayed ART initiation, such as development of AIDS conditions [40].

Preventing the secondary spread of HIV during the window period is an important public health preventive measure because of extremely high viral loads during this period [41]. Individuals with early HIV infection contribute significantly to the secondary transmission of HIV, and thus play a big role in sustaining new HIV infections [4,6]. Other stages of HIV infection also have a significant contribution to secondary transmission of HIV [6].

Undiagnosed or untreated HIV infection in pregnant women results in a high risk of vertical transmission of HIV, and this risk is much higher with early HIV infection [42,43]. Although prevention of mother to child transmission (PMTCT) programme has led to a remarkable reduction of vertical transmission of HIV in SA and other countries [43,44], more needs to be done to eliminate vertical transmission. Accurate diagnosis of HIV infection during antenatal care creates an excellent opportunity for initiation of PMTCT. Thus, strengthening HIV testing at the points-of-care would contribute towards further decrease and elimination of vertical transmission of HIV.

The high frequency of new HIV infections undermines the global efforts of eliminating new HIV infections. The first 90 of 90-90-90 UNAIDS target might not be properly assessed if there are many HIV-infected individuals that are misdiagnosed by rapid tests at points-of-care in resource-constrained settings. A test and treat strategy might not have a desired impact in low resource setting if point-of-care testing for HIV is not strengthened. Misdiagnosis of HIV infection could lead to inadvertent use of pre-exposure prophylaxis in HIV-infected individuals, thus resulting in emergence and transmission of ARV drug-resistant strains.

Detection of new HIV infections allows a close monitoring of the HIV epidemiology. This monitoring can be used to assess the effectiveness of the existing HIV diagnostic, treatment and prevention policies, and inform necessary changes to such policies.

### Possible explanations for misdiagnosis of HIV-infected people at the points-of-care

The finding that the majority of participants (95%) who were misdiagnosed at the points-of-care were positive on HIV ELISA is not surprising as the rapid tests have poorer sensitivity for the detection of HIV antibodies, especially during the early HIV phase [45]. In this study most participants with detectable antibodies had positive W. Blot and also tested positive on repeat rapid testing in plasma. The reason for false negative rapid tests at the points-of-care could be due to human errors during testing. These errors could include insufficient volume of blood and/or incorrect volume of diluent used [31].

Other researchers have previously showed inferior performance of some rapid HIV tests on finger-stick whole blood compared to testing done on serum in a setting where testing was done by skilled personnel [8]. Black et al. showed a remarkably lower sensitivity of rapid tests during a field evaluation done by trained nurses on finger-stick whole blood compared to the laboratory evaluation done on serum samples [36]. A recent study that screened for acute HIV infection in SA also detected a significant number of chronic HIV infections that were missed at the points-of-care [21]. The lower performance of some rapid tests in finger-stick whole blood could be caused by dilution effect from the red blood cells and/or weak antibody binding due to haemolysis [8], and by antigen-antibody complexes predominantly found in early HIV phase before the appearance of free antibodies [41]. These complexes are also found in varying degrees in later stages of HIV infection [46,47]. This shows a need to use rapid HIV tests with higher performance in finger-stick whole blood, as this is a sample type used at the points-of-care. One such test is INSTI, a rapid HIV test approved by the Food and Drug Administration (FDA), which has shown a high sensitivity and specificity in diagnosing HIV infection in whole blood samples [8]. The INSTI rapid test has also been reported to perform well in identifying individuals with early HIV infection [48].

The limitations of this study are that follow-up visits for NAAT-positive participants were done at different intervals and there was no active tracing of their partners. However, all participants identified with HIV infection were encouraged to disclose to their partners, and return with them to the clinics. Repeat rapid testing from plasma samples was not done before NAAT and serology testing. If done earlier, this would have led to faster identification of HIV-infected individuals as most rapid tests were positive in plasma samples.

## Conclusions

These data show that it is feasible to detect acute, early and chronic HIV infections initially missed by rapid tests in an HIV hyper-endemic setting with limited resources. The routine implementation of a 4 week follow-up rapid test would reduce both HIV diagnostic and transmission window periods. Rapid HIV tests with higher performance in finger-stick whole blood should be used in resource poor settings with high HIV prevalence. The newer molecular tests that can be used at the points-of-care should be evaluated for routine diagnosis of HIV in HIV hyper-endemic settings.
